# Dissociating COVID-19 from other respiratory infections based on acoustic, motor coordination, and phonemic patterns

**DOI:** 10.1038/s41598-023-27934-4

**Published:** 2023-01-28

**Authors:** Tanya Talkar, Daniel M. Low, Andrew J. Simpkin, Satrajit Ghosh, Derek T. O’Keeffe, Thomas F. Quatieri

**Affiliations:** 1 MIT Lincoln Laboratory, Lexington, MA USA; 2 Speech and Hearing Bioscience and Technology, Harvard Medical School, Boston, MA USA; 3 MIT McGovern Institute for Brain Research, Cambridge, MA USA; 4 HIVE Lab, School of Medicine, Lambe Institute, National University of Ireland, Galway, Ireland; 5 School of Mathematical and Statistical Sciences, National University of Ireland, Galway, Ireland

**Keywords:** Diagnosis, Computational biology and bioinformatics

## Abstract

In the face of the global pandemic caused by the disease COVID-19, researchers have increasingly turned to simple measures to detect and monitor the presence of the disease in individuals at home. We sought to determine if measures of neuromotor coordination, derived from acoustic time series, as well as phoneme-based and standard acoustic features extracted from recordings of simple speech tasks could aid in detecting the presence of COVID-19. We further hypothesized that these features would aid in characterizing the effect of COVID-19 on speech production systems. A protocol, consisting of a variety of speech tasks, was administered to 12 individuals with COVID-19 and 15 individuals with other viral infections at University Hospital Galway. From these recordings, we extracted a set of acoustic time series representative of speech production subsystems, as well as their univariate statistics. The time series were further utilized to derive correlation-based features, a proxy for speech production motor coordination. We additionally extracted phoneme-based features. These features were used to create machine learning models to distinguish between the COVID-19 positive and other viral infection groups, with respiratory- and laryngeal-based features resulting in the highest performance. Coordination-based features derived from harmonic-to-noise ratio time series from read speech discriminated between the two groups with an area under the ROC curve (AUC) of 0.94. A longitudinal case study of two subjects, one from each group, revealed differences in laryngeal based acoustic features, consistent with observed physiological differences between the two groups. The results from this analysis highlight the promise of using nonintrusive sensing through simple speech recordings for early warning and tracking of COVID-19.

## Introduction

Individuals affected by the disease COVID-19 (Corona Virus Disease of 2019), caused by the Severe Acute Respiratory Syndrome Coronavirus 2 (SARS-CoV-2), can have significantly different clinical presentations, from asymptomatic to severe illness^1^. Common symptoms of COVID-19, which may appear 2–14 days after exposure to the virus, include fever or chills, cough, shortness of breath, fatigue, new loss of taste or smell, a sore throat, and congestion or a runny nose^1^. As of April 2022, there have been over 523 million cases of COVID-19 across the world since its discovery in December 2019, which have led to global shortages of healthcare resources during the pandemic^2, 3^. This highlights an urgent need for the development of innovative technologies for screening, triage, and monitoring of infected individuals. In particular, there is a need to promptly identify and isolate patients with COVID-19 in a cost-effective manner to perform daily testing while maintaining social distancing to avoid the spread of the virus. Various forms of remote monitoring have used physiological sensors and signals for this purpose, and in particular, researchers have turned to speech and cough recordings to be able to develop models for screening and monitoring of COVID-19 using non-invasive methods. The goal of our work is to develop a set of speech-based biomarkers which not only help screen for and monitor COVID-19, but also aid in a physiological explanation for how COVID-19 affects speech production in infected individuals.

COVID-19 has been shown to present as a dysfunction in respiratory physiology, particularly affecting inhalation and exhalation of air from the lungs, which could affect speech production^4^. In particular, speech production requires the fine coordination of muscles within and across the articulatory, laryngeal, and respiratory subsystems^5–7^. Coordination involves the precise amplitude and timing of movements within and across these subsystems to reach desired speech targets. It has been found that movements within the respiratory subsystem are highly coordinated with movements within the laryngeal subsystem^5, 6^. The coordination of these movements aid in modulating the rate of speech, the presence of pauses for inhalations, the intensity of speech, the breathiness quality of speech, and the steadiness of fundamental frequency, or ‘pitch’, during phonation. In addition, laryngeal movements are tightly coupled with articulatory movements in the vocal tract^7^. Therefore, the hypothesis for speech and voice based analysis of COVID-19 is that the common symptoms of COVID-19 and their subsequent effect on the respiratory system will affect coordination of speech production subsystems, and will manifest as differences in speech acoustics. These differences can subsequently manifest in analysis of features derived from recordings of speech from individuals with COVID-19 compared with individuals without COVID-19.

Much of the work using acoustic data to detect COVID-19, specifically discriminating between individuals with COVID-19 versus healthy controls, relies on recordings of coughs, breathing, and short speech segments, such as sustained vowels. Utilizing Mel-Frequency Cepstral Coefficients (MFCCs), acoustic features which represent vocal tract articulator movements, extracted from cough recordings collected from individuals with and without COVID-19 through the OpenSigma project, LaGuarta et al. (2020) was able to obtain an area under the receiver operator characteristic (ROC) curve (AUC) of 0.97^8^. Samples collected in the CoughDetect initiative with Andreu-Perez et al. (2021) achieved an AUC of 0.98^9^. Classification using cough recordings collected through the Coswara initiative and DiCOVA challenges has been shown to achieve and AUC of 0.85^10^. Many of these studies utilize convolutional neural networks with MFCC spectrums as inputs. Though quite a few studies have focused on cough, there has also been promise shown for detection from speech recordings. Han et al. (2021) analyzed voice recordings from the University of Cambridge based COVID-19 Sounds App^11^. Utilizing a combination of acoustic based features and symptom information, they attained an AUC of 0.79. Data from a smartphone based collection from Sonde Health, using various short speech phrases, allowed Stasak et al. (2021) to detect individuals with COVID-19 with an accuracy of between 82 and 86%^12^. Pinkas et al. (2020) utilized sustained phonemes and a counting task to achieve an AUC of 0.81 when models formed from all of the tasks were combined into an ensemble model^13^. Saggio et al. (2021) utilized features from sustained vowels to discriminate between positive COVID-19 patients and healthy controls with an AUC of 0.94, and between positive -COVID-19 patients and recovered negative COVID-19 individuals with an AUC of 0.97^14^. While there has been promise in using these acoustic recordings, many of them utilize deep learning techniques that do not easily reveal the most important features, which could be used to better understand the physiological underpinnings of how speech is affected during COVID-19 and hopefully help generalize to future samples. This may also add to understanding why some speech tasks outperform others and provide further insight into the usefulness of different speech tasks for diagnosing and screening for COVID-19.

**Figure 1:**
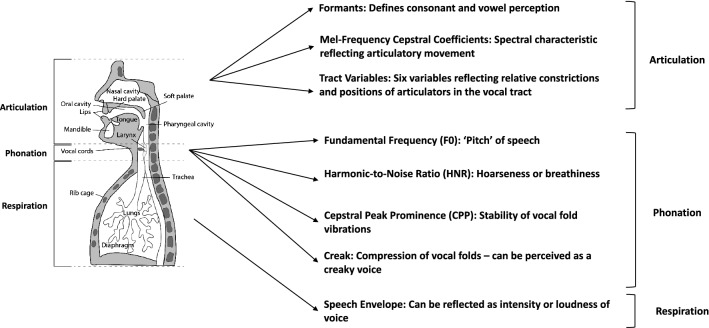
Speech production subsystems and representative acoustic features. Acoustic and articulatory features extracted from audio recordings, as well as their physiological and perceptual interpretations.

One approach to measuring the speech motor coordination differences between individuals with COVID-19 and individuals without COVID-19 is to utilize correlation structures derived from acoustic features extracted from speech recordings. These features have previously been used to predict and characterize motor difficulties in individuals with major depressive disorder (MDD)^15^, Parkinson’s disease^16^, and traumatic brain injury^17,18^. The analyses focus on the coordination within and across three major speech subsystems—articulatory, laryngeal, and respiratory. The subsystems, as well as the acoustic time series drawn from them, are depicted in Fig. 1. The correlation features have been used with a small set of data from five subjects to compare their speech before diagnosis of COVID-19 to their speech while they had COVID-19^19^. As compared to their pre-COVID-19 voices, the correlation-based features implied that there was more coupling and less independence of subsystem movement, particularly between and within the laryngeal and respiratory subsystems. This suggested that COVID-19 may change the motor coordination of speech production while individuals have the virus. Specifically, individuals are expected to have a certain degree of independence of their subsystems and muscles to allow for specific patterns of vocal fold vibrations and breathing, which may become more labored or require more effort when an individual has COVID-19, leading to the coupling observed. In addition to the coordination based features, phoneme-based features, looking at durations and rates of individual and groups of phonemes, have been used to provide insight into the motor ability of individuals with Parkinson’s disease by predicting scores on the motor component of the Unified Parkinson’s Disease Rating Scale (UPDRS)^16^. The coordination features derived from correlation structures as well as the phoneme-based features and univariate statistics of acoustic features representing the speech production subsystems can provide further insight into the effects of COVID-19 on speech production.

This study builds upon the earlier work done with five subjects^19^ and expands to a larger dataset collected at and beyond a hospital stay from COVID-19 positive patients and COVID-19 negative patients, specifically patients who had seasonal influenza or a viral infection such as respiratory syncytial virus (RSV) or Rhinovirus. Patients were asked to undergo a simple speech protocol with a set of tasks recorded daily on distributed iPads. The protocol consisted of a free speech prompt, a diadochokinetic sequence, a read passage (the Rainbow Passage^20,21^), a sustained vowel, and a counting task. From these recordings, machine learning models are generated utilizing correlation structure-based features, phoneme-based features, and univariate statistics of acoustic features to predict the COVID-19 status of the patients over three days of data collection. In addition, the best performing features and speech tasks are analyzed to help understand the physiological effects of COVID-19 on the speech production subsystems as compared to the physiological effects of a viral infection.

## Results

Participants included 28 patients at University Hospital Galway, Ireland who took part in a longitudinal collection of speech data during their stay at the hospital and recovery at home. The COVID-19 positive patient group consisted of 6 males with a mean age of 31.33 years (SD = 9.09) and 6 females with a mean age of 28.50 years (SD = 4.32), for a total of 12 patients. The COVID-19 negative patient group included 3 males with a mean age of 38.00 (SD = 18.35) and 12 females with a mean age of 36.83 years (SD = 5.41), for a total of 15 patients. A total of 27 patients, with their diagnosis verified by reverse transcription polymerase chain reaction (RT-PCR) tests, were included from the original 28 patients enrolled. All patients without COVID-19 were considered to have seasonal influenza or another viral infection, verified by doctors at University Hospital Galway.

We report the results for the acoustic, motor coordination, and phoneme-based models in turn, followed by a longitudinal case study.

### Models using low-level acoustic and articulatory features

A set of acoustic features were extracted, representing the three speech production subsystems, creating time series of the feature for each recording. In addition to the acoustic features, we also extracted time series of six tract variables, representing positions of the articulators in the vocal tract, for a more physiologically accurate representation of movement in the vocal tract^22^. For each feature time series, we computed the mean and standard deviation as input features for a gaussian mixture model (GMM) to detect the presence of COVID-19 in individuals. The models were trained utilizing a bootstrapping approach, with separate models created for each task. In addition to a model trained and tested on the data collected on the first day of collection, with all 27 individuals, models were trained on the data collected on the first day, and tested on the data collected by individuals on the second or third session of recording.

**Figure 2:**
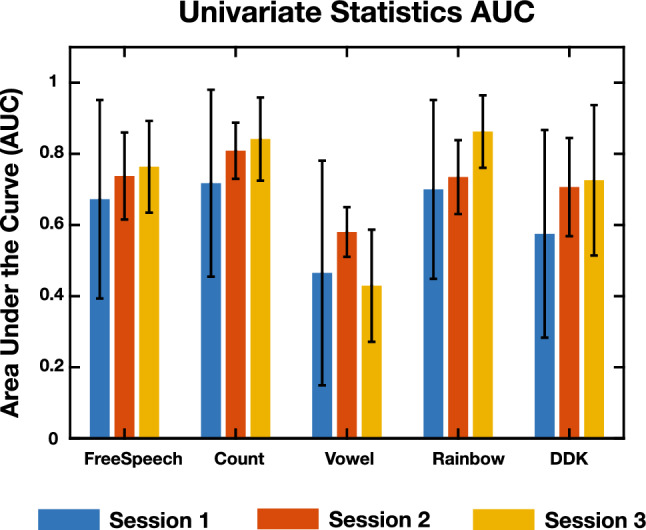
Model performance using low-level acoustic features. AUC scores from models created using univariate statistics of acoustic and articulatory features. The different bars in each task represent which of the recording days was used in the test data set. Overall, for most tasks, performance increased on predicting COVID-19 status on subsequent days of recording. Statistics derived from the Count task appear to outperform the other tasks when used in a predictive model. Error bars are the standard deviation of the statistic across all bootstrap iterations.

Figure 2 shows the mean AUC over all bootstrap iterations using the acoustic and articulatory features for each of the tasks. This is for predicting the presence of COVID-19 for both the first session, but also the prediction for the 2nd and 3rd recording sessions. The error bars in these plots, as well as in subsequent plots, represent the standard deviation of the statistic across all of the bootstrap iterations. Supplementary Fig. S2 shows the accuracy and F1 scores of the models.

Looking at solely the performance using the first day of recording, the univariate features derived from the Rainbow task, AUC of 0.75, performed significantly better than the Free Speech, Vowel, and DDK tasks, with exact *p*-values provided in Supplementary Table S2. The model derived from the Count task also had an averaged AUC of 0.75, but was only significantly greater than the models derived from the Vowel and DDK tasks (Supplementary Table S2).

When looking at the predictive value of a model trained on data from the first day, predicting the COVID status of subsequent days, the Count task stands out as the task which leads to high performance, with an AUC of 0.80 for Day 2 and 0.84 for Day 3 (significance shown in Supplementary Table S3). The Rainbow task specifically has good performance on predicting Day 3, with an AUC of 0.86, and was significantly greater than all univariate models except Count Day 3 (Supplementary Table S3). The Vowel task, however, shows a decrease in performance on Day 3 (Supplementary Table S3), which may have to do with the fewer number of subjects being evaluated or perhaps more variability in sustained vowel performance as individuals recover. Overall, the performance improved across most tasks, showing that building the model on the information from an early recording seems to help in predicting outcomes on subsequent days, suggesting that the symptoms stay constant during the acute phase of the illness.

### Correlation-based motor coordination features

GMMs were created using the correlation-based features derived from correlations of acoustic time series and their deltas across and within speech production subsystems, leading to a total of 34 models per task per session. Given the number of correlation features that were extracted, the top three performing features in each task, comparing the model generated on the first day’s data, were isolated for comparison.

**Figure 3:**
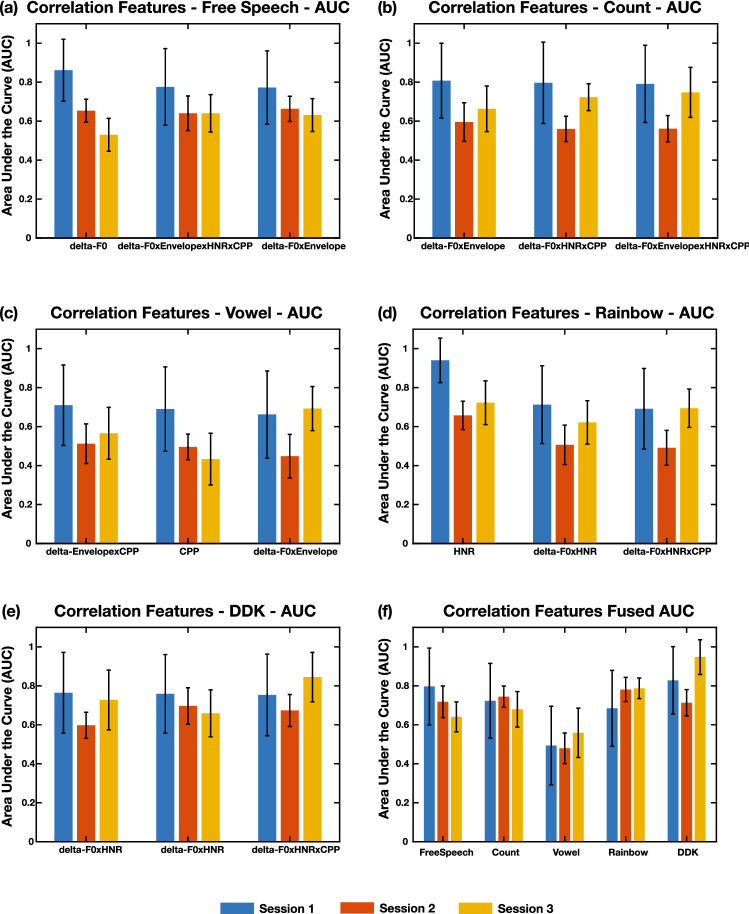
Top correlation-based features’ AUCs across tasks and fused model performance. (**a**–**e**) AUC scores across prediction of three different days for the top three performing features in each of the five. The features appear to be most commonly from the laryngeal subsystem. HNR during the Rainbow task has the highest AUC at 0.94. (**f**) AUC scores from models created by fusing across all models created from the correlation based features. There is a decrease seen in performance relative to the top feature in the Free Speech, Count, Vowel, and Rainbow tasks, and an increase in performance relative to the top feature seen in the DDK task. Error bars are the standard deviation of the statistic across all bootstrap iterations.

Table 1 gives the top three performing features in each task, evaluated by looking at the performance of the models created on the Day 1 data. Figure 3a–e show the AUCs of these features over all three days. The full table of comparisons of p-value significance to determine the top features is presented in Supplementary Data 1. While the top three features are shown here for ease of display, the full set of features and their AUCs for each task, as well as the AUCs of all bootstrap iterations, are located in Supplementary Data 2.

**Table 1:** Top correlation-based features. Top 3 performing features in each task in creating GMM models using correlation-based features. The top 3 features were selected by comparing the performance of the models trained and tested on the recordings from the first day.

	1st Feature	2nd Feature	3rd Feature
Free Speech	Delta-F0	Delta-F0xEnvelopexHNRxCPP	Delta-F0xEnvelope
Count	Delta-F0xEnvelope	Delta-F0xHNRxCPP	Delta-F0xEnvelopexHNRxCPP
Vowel	Delta-EnvelopexCPP	CPP	Delta-F0xEnvelope
Rainbow	HNR	Delta-F0xHNR	Delta-F0xHNRxCPP
DDK	Delta-F0xHNR	Delta-F0xCPP	Delta-F0xHNRxCPP

The majority of the top correlation-based features are comprised of the delta features that represent the laryngeal and respiratory systems. In particular, deltas of F0, HNR, CPP, and the envelope show up in the top features. There are exceptions of non-delta based features with the high performance of HNR during the Rainbow task, with an AUC of 0.94, and the inclusion of CPP during the Vowel task, with an AUC of 0.69. This suggests that many of the detectable differences between COVID-19 positive individuals and COVID-19 negative individuals may show up in the laryngeal and respiratory subsystems during speech production. This appears to be consistent with the physiological effects of COVID-19, which primarily is affecting the respiratory system, and therefore may affect the movements of the laryngeal subsystem, given the tight coupling between the two. For many of the tasks and features, there seems to be a decrease in performance when predicting COVID-19 status on the second and third days of data collection.

In addition to looking at individual features, a fused model across all of the correlation-based features within a task was computed to assess how the features could complement each other in the creation of a model. Figure 3f shows the AUC across all three days of the fused model for each task. Supplementary Fig. S3 shows the accuracy and F1 scores of the fused model of each task.

An increase in performance over the top performing feature in the task was seen in the DDK task (AUC = 0.83; *p* = 0.01), while a decrease was seen in the Free Speech (AUC = 0.80; *p* = 0.002), Count (AUC = 0.72; *p* = 0.001), Vowel (AUC = 0.49; *p* = 7.74 × 10^–11^), and Rainbow (AUC = 0.68; *p* = 1.08 × 10^–15^) tasks. This may indicate the differing demands of the tasks and differing effects of COVID-19 on performance on the tasks. In particular, the addition of the articulatory features may aid in detection for the DDK, due to the high articulatory demands of the task. However, given the variability of articulatory demands in the Free Speech, Count, and Rainbow tasks, which may lead to more degrees of freedom of movement for the prescribed tasks, the model performance may not benefit from the addition of the articulatory information. In addition, the stability in articulatory movements required in the Vowel task may not have differed much between the two groups, leading to lower performance when articulatory features were added in fusion.

### Phoneme-based features

Just as with the univariate statistics and correlation-based features, models were generated for sets of the phoneme-based features. Separate models were generated for three of the tasks using four different sets of the features: (1) all features (1216 total features reflecting univariate statistics and other statistics related to the phoneme durations), (2) mean durations of all phonemes, (3) rates of all phonemes, (4) a set of 6 summary statistics, including the articulation rate (# phonemes/total duration of recording—total duration of pauses), the speech rate (#phonemes/total duration of recording), the total duration of the recording with and without silence, the total number of phonemes, and the total number of unique phonemes. The models for the phoneme based features were only generated for the free speech, count, and rainbow tasks given the higher diversity of phonemes included in those tasks. As with the other sets of features, models were generated for predicting COVID-19 status on the first three sessions.

**Figure 4:**
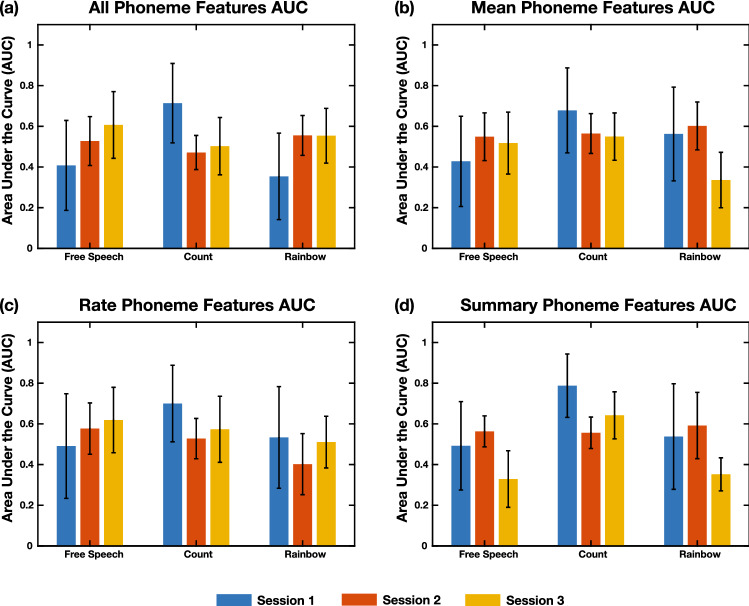
Model performance utilizing phoneme-based features. (**a**) AUC scores from models created using all phoneme-based features. The different bars in each task represent which of the recording days was used in the test data set. Statistics show that the phoneme based features generally don’t seem to outperform the univariate statistics. (**b**) AUC scores from models created using the means of the phoneme durations. Overall, these features used to train a model seem to perform similarly to using all of the phoneme based features across all days. (**c**) AUC scores from models created using the rates of the phonemes. The rate features seem to perform similarly to using all of the phoneme based features and just using the means of the phoneme durations. (**d**) AUC scores from models created using a set of six summary statistics of the phoneme based features. Features derived from most of the tasks seem to perform similarly to the other sets of features selected for model creation. Error bars are the standard deviation of the statistic across all bootstrap iterations.

Figure 4a shows the mean AUC over bootstrap iterations using all of the phoneme based features that were extracted. Because the Kaldi model was not explicitly trained on diadochokinetic speech, and there is only a single sustained phone expected for the Vowel task, those tasks was excluded from model creation and evaluation. The figure is comprised of the performance on the subsequent sessions 2 and 3 as well.

Figure 4b shows the performance using the mean duration of phonemes, and Fig. 4c shows the performance using the rates of the phonemes (# of occurrences of the phone/ total duration of all of the occurrences of the phone). Figure 4d shows the performance using the set of 6 summary statistics. Supplementary Tables S4, S5, S6, and S7 show the significant differences between the models within each phoneme-based feature category along with the *p*-values. The full table of comparisons of significances is presented in Supplementary Data 1. Supplementary Figs. S4, S5, S6, and S7 additionally provide the accuracy and F1 scores for all four categories of phoneme-based features.

Models derived from the phoneme based features from all tasks in general seem to have lower AUCs as compared to models generated with univariate statistics of the acoustic and articulatory time series. In particular, the AUCs for many task and feature combinations are below 0.5, indicating poor detection of COVID-19. This may indicate that the phoneme features themselves, with regards to phoneme durations and rates, may not be changing for individuals with COVID-19.

When predicting COVID-19 status on subsequent dates, using all of the phoneme features and the mean phoneme durations leads to a dip in performance for the Free Speech task on the 3rd day. There is an increase in performance on the 3rd day for the Count task for all of the phoneme feature subsets, except the summary statistics. Other comparisons were less consistent across feature sets and days of prediction. However, as shown in the *p*-values provided in Supplementary Data 1, many of the phoneme-based feature models had lower AUCs than the univariate statistics and correlation-based feature models, suggesting the phoneme statistics may be less useful in discriminating between the two groups.

### Case study

**Figure 5:**
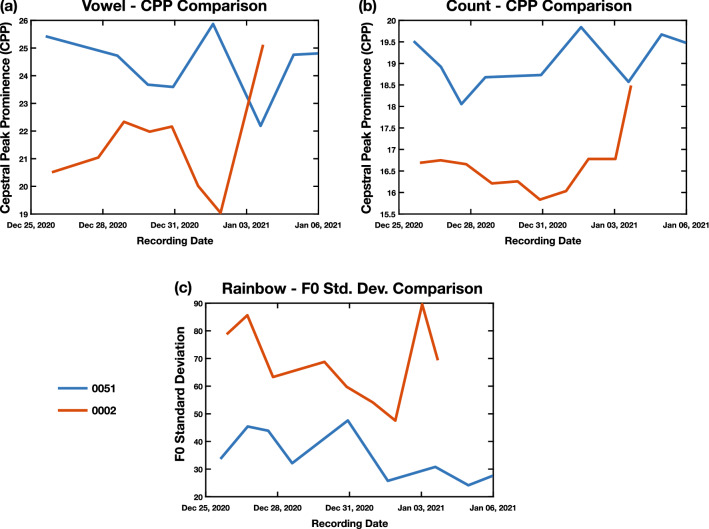
Case study comparisons of CPP and F0 between subjects 0051 and 0002. (**a**) CPP values over time for subjects 0051 (COVID-19 negative) and 0002 (COVID-19 positive) for the Vowel task. We see an increase in CPP at the end of the recording period of Subject 0002, reaching a CPP level which is close to the CPP value of Subject 0051. (**b**) CPP values over time for subjects 0051 and 0002 for the Count task. We similarly see an increase in CPP at the end of the recording period of Subject 0002, leading to a CPP value close to that of Subject 0051. This suggests that vocal fold vibration stability is increasing as Subject 0002 is recovering in both tasks. (**c**) This figure plots the values of the standard deviation of F0 during the Rainbow task for subjects 0051 (COVID-19 negative) and 0002 (COVID-19 positive). The standard deviation for Subject 0051 is lower than that of Subject 002, suggesting more stability in F0 contours during the passage.

Two participants, one COVID-19 positive (Subject 0002) and one COVID-19 negative (Subject 0051) participated in the study for 10 days each. Subject 0002 participated from 12/26/2020 to 1/4/2020, recording every day. Subject 0051 participated from 12/26/2020 to 1/6/2020, missing two days in between. These longitudinal recordings allowed for assessment of changes throughout the recording period as well as longer comparisons between the two subjects. Figure 5 describes some of these observations.

Figures 5a,b contain the comparisons of the CPP values during the recording period for the Vowel and Count tasks. In both figures, we see an increase in CPP near the end of the recording period for Subject 0002, coming closer towards the CPP values of 0051. A higher CPP value indicates more regularity in vocal fold vibrations, and therefore healthier vocal folds, so this suggests that the effect that COVID-19 has on the laryngeal system may be lessened as Subject 0002 is recovering, though they are still testing as positive.

Figure 5c shows the comparison of the standard deviation of F0 during the Rainbow task across the recording periods of the two subjects. The standard deviation of F0 for Subject 0051 is lower than the standard deviation for Subject 0002 across the recording period, suggesting that there is more stability in F0 in Subject 0051. This may indicate that COVID-19 has an effect producing a stable F0, which explains why it manifests as an important feature in the coordination-based analysis.

## Discussion

This paper presents a study assessing the effects of COVID-19 on speech production through an analysis of motor coordination-based features, univariate statistics of acoustic time series, and phoneme-based features. Features and tasks were chosen to assess the effects on the three main speech production subsystems—articulatory, laryngeal, and respiratory. Eigenvalues derived from correlation structures of time-series of acoustic and articulatory features were able to discriminate between individuals with COVID-19 and those who tested negative for COVID-19 but had another viral respiratory disease with an AUC of 0.75 using univariate statistics derived from the Rainbow and Count task and 0.94 using HNR coordination-based features derived from the Rainbow task. These tasks were specifically chosen to tax the laryngeal and respiratory subsystems, as the Rainbow task asked subjects to perform the task in one breath, while the Count task asked subject to perform the task as quickly as possible. Furthermore, for some combinations of features and tasks, there was similar, if not better, model performance seen when predicting the status of the patients on subsequent days of data collection. This indicates that a multitude and variety of acoustic-based features show promise in discriminating individuals who are COVID-19 positive from those with seasonal influenza, and specifically that there may be features sourced from the laryngeal and respiratory speech production subsystems that aid in discrimination.

There were differences observed in the discriminative ability of features across different tasks, indicating that the tasks are providing different insights into the effect of COVID-19 on speech production. In particular, the Rainbow task stood out when creating models from univariate statistics, outperforming the other tasks. Across all feature sets, however, the Vowel task did not appear to perform very well as compared to the other tasks, suggesting that for these features, there is not enough of a difference witnessed in the speech production demands needed to sustain a vowel, as compared to the additional demands that would be needed in the other tasks.

Within the correlation-based features, the top performing features were mostly based on cross-correlations between laryngeal and respiratory subsystem features. This appears to be consistent with the physiological effects of COVID-19, indicating that the physiological manifestations of COVID-19 may affect motor coordination between and within the laryngeal and respiratory subsystems. In particular, the deltas of the features showed up as common top features, indicating that the differences may lie in the velocity in the movements of the underlying muscle groups. In addition, features derived from the articulatory subsystem, such as MFCCs, which to date have been most commonly used in the generation of machine learning models to detect COVID-19, did not perform as well. However, for the DDK task, it was found that fusion using coordination features derived from all three subsystems increased performance relative to using individual features. This will need to be investigated further to better understand and interpret the use of MFCCs in detection of COVID-19 and how we may be able to augment existing, high performing models with laryngeal and respiratory based features.

In the use of the correlation-based features, it will be important to further understand and quantify the additional information that they provide past what is accessible through univariate statistics or use of the time series themselves. While the correlation-based features have been used to date as a proxy for motor coordination, our future work aims to quantify how changes in the frequency spectrum of the underlying time series are reflected in the eigenvalues derived from correlation matrices. In addition, just as we utilized acoustic-to-articulatory inversion to derive more physiologically accurate movements of the articulators, it will be important to look into signal processing techniques which allow for the derivation of physiologically accurate representations of the laryngeal and respiratory subsystems as well.

In future phases of this work, we aim to validate the patterns observed here with a larger group of individuals. Additionally, we aim to better understand the differences between individuals with COVID-19 and individuals with a variety of respiratory-based illnesses. We hope to compare features that have been isolated here as showing high performance to those that may aid in detecting individuals who have other respiratory illnesses to determine if they are affecting different interactions between or within the subsystems. In addition, it may be that individuals with other respiratory illnesses may show differences in speech production across tasks as compared to individuals with COVID-19, as was observed here with other viral respiratory infections. The analysis of individuals who have one of the many variants of COVID-19, as well as the “breakthrough” cases that occur in individuals who have been vaccinated may also provide further insight into how the virus is evolving with regards to its physiological effects. The development of the non-invasive recording platform used in this paper aids in the deployment of data collection not only across hospitals such as the University Hospital Galway, but also in communities where standard testing is not easily accessible, and can aid in collecting patients with other illnesses.

Furthermore, we plan to collect additional longitudinal data to be able to track and monitor the changes observed in the features used as individuals recover from COVID-19. Observing the change over a longer period of time may also aid in a better understanding of the physiological effects of COVID-19 on speech production. This longitudinal tracking could also be used in at-home settings to determine if individuals need to come in for additional interventions or hospitalization, or, in the hospital setting, could be used to augment existing tests for discharge. As alluded to earlier, this longitudinal data could also be compared across individuals with different variants or different respiratory illnesses to determine how recovery trajectories change across these variables.

## Methods

### Subject recruitment

Patients with symptoms of respiratory illness from the University Hospital Galway (Ireland) were recruited between December 2020 and May 2021 and asked to participate in a longitudinal collection of speech data during their stay at the hospital and recovery at home. The COVID-19 diagnosis of all subjects was verified by reverse transcription polymerase chain reaction (RT-PCR) test. All individuals without COVID-19 were considered to have seasonal influenza or another viral infection, diagnosed by doctors at the hospital, and with symptoms that were clinically serious enough to warrant hospital review and admission. One subject was excluded due to their unverified COVID-19 diagnosis, as their test results had not been reported at the time of collection. The ratio of 12 COVID-19 positive subjects and 15 COVID-19 negative subjects was shown to have an adequate power of 0.80 with a Type 1 error rate of 5% to detect differences between AUCs of the models generated from a pilot study with a subset of this subject group. All subjects were unvaccinated, and the predominant strain at the time of data collection was the alpha strain, though participants were not specifically tested for the strain. While specific race and ethnicity information was not collected from each individual, 91% of individuals in Galway are Caucasian Irish, and all subjects had the regional accent^23^. The procedures were approved by the University of Galway, and MIT’s Committee on the Use of Humans as Experimental Subjects Institutional Review Board additionally approved the voice data collection. All subjects signed an informed consent prior to the participation in the study. The experimental protocol was performed in accordance with the guidelines and regulations of the two institutions.

### Experimental protocol

All recordings were collected using a COVID-19 version of the Reproschema platform (https://www.repronim.org/reproschema/),^24^ which guided the subjects through three sets of surveys on an iPad. The full protocol is openly accessible (https://github.com/sensein/covid19). Each participant was given a unique identifier to log in to the platform. The participants were asked to record data for every day of their stay in the hospital, up to 14 days. Not all participants recorded longitudinal data. 6 individuals who were COVID-19 positive and 10 individuals who were COVID-19 negative, a total of 16 individuals, recorded at least two days’ worth of data. 4 individuals who were COVID-19 positive and 5 individuals who were COVID-19 negative, a total of 9 individuals, recorded at least three days’ worth of data.

The first survey collected demographic information, information about the participant’s COVID-19 status, information about their smoking history, and a place to report any health related symptoms, such as a cough or fever. T-tests were conducted with the demographic information to ensure there was no risk of bias in model development. *P*-values were insignificant for differences in age (*p* = 0.7994), smoking status (*p* = 0.2030) and gender (*p* = 0.1081). There was a significant difference in the number of symptoms that each group reported (*p* = 0.000046), with the COVID-19 positive group reporting a higher number of symptoms. However, there were more reports of myalgia in the COVID-19 negative group (*p* = 0.0002), which matches other reports of symptoms in COVID-19 positive patients versus COVID-19 negative patients^25^. More information about the symptoms reported and demographic information is provided in Supplementary Table S1.

The second survey consisted of the audio data collection. The participants first performed an audio check to ensure that they were not in a location which had too much noise. From there, the platform guided the participants through the protocol using prompts. The tasks were chosen to capture various breathing and articulation patterns in different contexts. To record the audio, participants would press the Record button before speaking, and then the Stop button once they were done. They were instructed to use their typical volume and typical pitch during speaking tasks. The first speaking task was a Free Speech task, where participants were instructed, “Tell me how you’re feeling, and why you think you feel that way with as much detail as possible.” And were given up to three minutes to speak. Next, in the Counting task, participants were instructed, “Using your normal voice, count upwards from 65 to 105 as fast as you can.” This task specifically was used to tax the individual’s use of nasals and fricatives for breathing and use of the respiratory system. Subsequently, for the Sustained Vowel task, the participants were instructed, “Using your normal voice, take a deep breath and say \ah\ until you run completely out of air. This task was expected to last for approximately 5–20 s. Then for the Rainbow Passage task, the participant was asked to use their normal voice to read a shortened version of the Rainbow passage^20, 26^, consisting of the first four sentences. They were directed to “Take a deep breath and read as much as you can in one breath using your normal voice (and stop when you run out of air)”. This was done to tax the respiratory system to potentially amplify differences between individuals with and without COVID-19. For the last of the speech based tasks, the Diadochokinetic (DDK) task, the participant was prompted to take a deep breath and repeat the phrase “pa-ta-ka” as many times as they could in one breath. The last task asked the participant to inhale, cough 3 times, and repeat that process two more times. The coughing task was not utilized in analysis to focus on speech production.

The third survey—not used in this study—was used to collect non-diagnostic information related to the participant’s current mental health status. The survey combined the Patient Health Questionnaire (PHQ)-2^27^, which seeks to screen for depression, and the General Anxiety Disorder (GAD)-7 assessment^28^.

For each session on the iPad for the participant, all of the information from the first and third survey were stored in JSON-LD files indicating the mapping between the question and the survey response. The unique names of the audio files recorded were also stored in a JSON-LD file containing the link between the type of audio file and the name of the audio file for retrieval. Audio files were sampled at a sampling rate of 48 kHz and recorded on distributed identical iPads.

### Low-level acoustic feature extraction

For the purposes of the analysis described here, low-level acoustic features were extracted from the Free Speech, Counting, Sustained Vowel, Rainbow Passage, and DDK tasks. These low-level acoustic features were chosen to represent the three subsystems of speech production and to provide an interpretable understanding of potential changes to the speech production subsystems if a participant is diagnosed with COVID-19. The full set of low-level features, their relation to specific speech production subsystems, along with their physiological description, is summarized in Fig. 1.

To represent the articulatory subsystem, we extracted vocal tract resonances (formants), mel-frequency cepstral coefficients (MFCCs), and tract variables (TVs). The first three formant time-series (F1-F3) were estimated using the Kalman-based autoregressive moving average (KARMA) software tool, calculated at 100 Hz^29^. KARMA utilizes an energy-based speech activity detector which is used for a Kalman smoother to extrapolate formants through silent gaps in the signal where no speech is being produced, allowing for continuous time-series of formants. MFCCs were extracted using the Praat toolbox^30^. The toolbox extracted 13 MFCCs using a window length of 5 ms and a time step of 5 ms, leading to time series at 200 Hz. We used acoustic-to-articulatory inversion software to extract six tract variables, or vocal tract constriction variables, following the Task Dynamics model: Lip Aperture (LA), Lip Protrusion (LP), Tongue Body Constriction Location (TBCL), Tongue Body Constriction Degree (TBCD), Tongue Tip Constriction Location (TTCL), and Tongue Tip Constriction Degree (TTCD)^22,31^. The software takes in an acoustic speech signal, extracts out MFCCs using the HTK software, uses a Vocal Tract Normalization technique to account for speaker variability, and utilizes a neural network to output the trajectories of the TVs at 100 Hz. The TVs allow for a more physiologically accurate representation of the movements within the articulatory subsystem as compared to MFCCs and formants and have been used in detecting motor coordination patterns in MDD^32,33^.

The laryngeal and respiratory subsystems were represented by features that provide insight into voice quality, prosody, and loudness. Fundamental frequency (F0) was extracted using the Praat toolbox at 1000 Hz^30,34^. A minimum F0 of 50 Hz and a maximum F0 of 300 Hz was used to constrain the values to physiologically relevant values, as well as to prevent pitch doubling in the output Praat trajectories. Harmonic-to-noise ratio (HNR) was also extracted using the Praat software at 100 Hz, representing the relative degree of breathiness and additional noise present from the laryngeal system during speech^34^. Cepstral peak prominence (CPP), representing the relative stability of vocal fold vibrations, was extracted using a custom-built script in MATLAB, based on the technique described in Awan et al. (2010)^35–38^. Creak, another measure of voice quality usually associated with vocal fry, was also extracted using a custom script in MATLAB based on the methodology described in Drugman et al. (2014)^39,40^. HNR, CPP, and creak were all extracted at 100 Hz. To represent the interaction between the laryngeal and respiratory subsystems, and specifically the contribution of the respiratory subsystem to speech, we extracted the speech envelope at 1000 Hz. This was done using a custom MATLAB script that provides a smooth contour of amplitude peaks based on an iterative time-domain signal envelope estimation^41,42^. The algorithm estimates both the contributions of the respiratory muscles and resonance-harmonics interaction to the amplitude modulation of a speech envelope.

Each audio task was treated as an individual segment from which to extract each of these time series. In addition to the time series, delta-time series were calculated for each of the features. Univariate statistics of mean and standard deviation were extracted from each time series for all task and feature combinations. These low-level acoustic time series were also subsequently used in extraction of *high-level* coordination features.

### High-level correlation-based feature extraction

**Figure 6:**
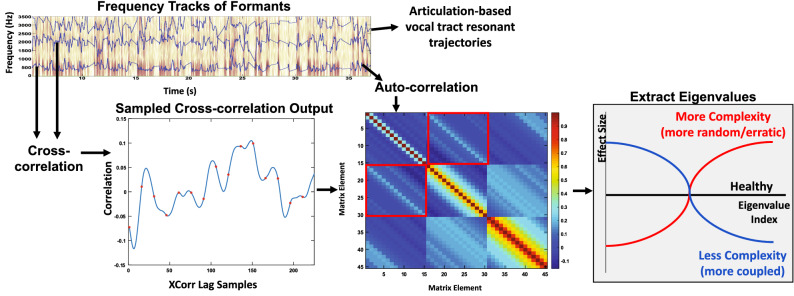
Correlation-based feature extraction. Process of extracting correlation based features through a correlation matrix technique. The patterns of eigenvalues provide insight into the relative complexity of the underlying signals.

Proxy measures of coordination within and across the underlying motor systems were calculated using multivariate auto- and cross-correlations of the low-level acoustic and articulatory features^43,44^. This process is described in Fig. 6. The time-delay embedding space of the low-level acoustic and articulatory time series is represented through the construction of channel-delay correlation matrices which expand the dimensionality of the low-level time series. The correlation matrices represent coupling strengths across feature channels at multiple relative time delays. The delay scales were delay spacings of 10, 30, 70, and 150 ms with 15 time delays used per scale to capture patterns at small time scales and also at the larger, phoneme level. From the eigenvalues of the correlation matrices, we can derive insights into the relative complexity of the underlying signals. Higher complexity across multiple channels of a feature or multiple features is represented in a more uniform distribution of eigenvalues, as opposed to lower complexity represented through a larger proportion of the overall signal variability being concentrated in a small number of eigenvalues.

Correlations across features were calculated within and across speech subsystems to capture the dynamics of motor coordination during speech. Feature combinations (e.g. F0 correlated with formants) were calculated by concatenating the feature vectors. For example, calculation of the correlation matrix for F0 and formants stemmed from a 4 * t feature matrix, where *t* is the total number of samples in the time series, due to a 1 * t F0 matrix concatenated with a 3 * t formant matrix. The full set of feature combinations that were used to calculate correlation matrices is listed in Table 2. This included both the extracted features and the delta-time series, for a total of 36 feature combinations. Any feature correlated with F0 was interpolated to a sampling rate of 1000 Hz using spline interpolation in MATLAB. Additionally, a masking technique was used for each correlation to include only the voiced segments in the correlations for each recording, using the locations where F0 > 0. We only looked at the correlations within the acoustic features, and did not include the articulatory tract variables in the cross subsystem correlations.

**Table 2:** Acoustic features used to build coordination features. List of features and combination of features that were used to assess coordination differences within and across speech production subsystems. Deltas of each of the entries below were also used to assess the correlations and coordination across feature velocities.

Base features	Cross subsystem correlations
Formants	F0 x Formants
Mel-Frequency Cepstral Coefficients (MFCCs)	F0 x Envelope
Fundamental Frequency (F0)	Envelope x Formants
Envelope	F0 x Envelope x Formants
Harmonic-to-Noise Ratio (HNR)	F0 x CPP
Cepstral Peak Prominence (CPP)	F0 x HNR
Creak	F0 x HNR x CPP
Tract Variables	F0 x HNR x CPP x Envelope
	HNR x CPP x Envelope
	CPP x Envelope

Eigenvalues of all resulting correlation matrices for feature combination and task were extracted by rank order (from greatest to smallest). Each correlation matrix was shaped (15 * n) x (15 * n), where n was the number of time series in the input matrix. This yielded 15 * n eigenvalues from each correlation matrix to comprise the eigenspectrum for a single delay scale (e.g. correlations of formants led to 15*3 = 45 eigenvalues). Eigenvalues from each delay scale for a single task and feature combination were concatenated to form a final feature vector with n * 15 * 4 elements to input into a classifier.

### Phoneme extraction

We utilized the Kaldi automatic speech recognition ASpIRE Chain Model, a model trained on Fisher English, to extract out automatically detected phonemes and phoneme durations from each of the speech recordings^45^. The automatically created transcripts contained the detected start and end time of each phoneme. From these transcripts, we extracted out univariate statistics related to the duration of each phoneme, as well as the rate of each phoneme or groups of phonemes. The rate was calculated as the number of instances of the phoneme or phoneme group divided by the total duration of all of the instances of the phoneme or phoneme group. These groups were also used to extract out articulation rate, which is the total number of phonemes divided by the duration of speech excluding silence, and speech rate, which is the number of phonemes divided by the duration of speech including silence. There were a total of 1216 phoneme-based features extracted. These statistics were used in various combinations to input into a classifier to assess for differences between individuals with COVID-19 and individuals without COVID-19.

### Classification

Classification of participants into individuals with COVID-19 and those without COVID-19 was done using a gaussian mixture model (GMM) trained on either a vector of univariate statistics (mean and standard deviation) derived from the low-level acoustic and articulatory time series, eigenvalues extracted from correlation matrices, or phoneme-based features. A separate GMM was created for each task and feature combination using a nested bootstrapping approach described in the next paragraph. This bootstrapping approach allows derivation of an unbiased estimate of model performance with the dataset available such that it is more likely to generalize to the features of the population, given averaging over multiple iterations of train and test sets. A description of this methodology is provided in Supplementary Fig. S1. The inputs into the GMMs were the features reduced in dimensionality using principal component analysis (PCA) with the optimal number of principal components for each model selected through bootstrapping on the training set. After starting with an ensemble of 10 GMMs created over four iterations with the training data (after PCA), COVID-19 positive and COVID-19 negative GMMs were generated using a supervised adaptation technique, which is a technique commonly used in speaker verification^46^. The likelihood of any test subject belonging to each group was computed by summing up the likelihood of the participant belonging to each GMM in the adapted ensemble. The final model output score was the ratio of the log likelihood of the participant belonging to the COVID-19 positive GMMs over the log likelihood of the participant belonging to the COVID-19 negative GMMs. The model output scores were used to compare model performance to determine the optimal number of principal components (PCs) to use, and to compare relative performance across features and tasks using the area under the receiver operating characteristic (ROC) curve (AUC), which is found by plotting the true positive rate against the false positive rate at various threshold settings.

The first set of models were trained and tested on data from the first day of collection from each of the participants. In each outer bootstrap iteration, a balanced set of 3 COVID-19 positive individuals and 3 COVID-19 negative individuals were held out as a test set such that there was no overlap between the training and test set and consequently no possibility of identifying the subject (speaker) rather than the viral condition. Within the training set, a set of 100 bootstrapping iterations was run to determine the optimal number of principal components to use. This involved holding out balanced sets of 3 COVID-19 positive individuals and 3 COVID-19 negative individuals selected from the training set, and calculating the average AUC obtained over all bootstrap iterations for a particular number of PCs. The number of PCs yielding the highest average AUC across all bootstrap iterations was chosen to be applied to the full training test. The COVID-19 positive and COVID-19 negative GMMs were created using the entire training set dimensionality reduced to the chosen number of PCs. From there, the AUC was calculated on the held out test set, as well as the accuracy and F1 score for the test set based on the optimal cutoff point on the ROC curve formed by the training set. This was repeated 99 more times for a total of 100 bootstrap iterations. The final model performance was reported for a specific task and feature combination using the average AUC, accuracy, and F1 score across all bootstrap iterations. Bootstrapping was used as the test sets in k-fold or leave-one-subject-out cross validation may not be as representative of the population or data in small datasets^47^.

A second and third set of models was created in a similar manner by training on data from the first day only, and then testing on either the recordings from the second or third day. In this case, the methodology was largely the same as described above, a set of 3 COVID-19 positive individuals and 3 COVID-19 negative individuals were left out on each bootstrap iteration, and the rest were used as the training set. The selection of the number of PCs to use was conducted in the same way as above, holding out balanced sets of 3 COVID-19 positive individuals and 3 COVID-19 negative individuals selected from the training set. The actual test set was selected from the participants who had recordings on the second or third days. There was no overlap between the individuals included in the training and test sets, even though they were from different days. There were fewer individuals who had recordings on these days. Performance for a single bootstrap iteration was reported as the AUC, accuracy, and F1 score on the test set with the model created on the training set for that iteration. Final performance was reported as the average AUC, accuracy, and F1 score over all bootstrap iterations. All individuals recorded their second or third sessions within a week of the first session, hence performance is expected to stay relatively constant across the sessions, since the individuals with COVID-19 were still in the contagious period.

A final set of models were created by fusing across all of the correlation based features derived from the first day recordings. In this case, a set of 36 models was created during each bootstrap iteration using the same test set. The model output scores for a test subject from each model were summed and then divided by 36 and were used as a final model output score to derive the model performance statistics of AUC, accuracy, and F1 score. These scores were then averaged across all bootstrap iterations to determine the final performance of the fused model across all of the correlation features for a single task.

When comparing models, the statistical significance of observed differences between AUCs was determined using the harmonic mean *p*-value methodology^48^. A model’s averaged AUC was considered to be greater than another model’s averaged AUC if the p-value resulting from a one-sided Wilcoxon rank sum test and the harmonic mean p-value was less than the significance threshold of α = 0.05. The Wilcoxon rank sum test was utilized under the assumption that the distribution of AUCs from one model was independent from the distribution of the AUCs from any other model, as well as the assumption that the variances of the AUCs in each model were equal. With a total of 606 models generated across all feature sets, we calculated a harmonic p-value of 1.11 × 10^–16^.

## Supplementary Information

Supplementary Information 1.

Supplementary Information 2.

Supplementary Information 3.

## Data Availability

The datasets analyzed during the current study are available from the corresponding author on reasonable request.
