# Supplementary material for: Dissociating COVID-19 from other respiratory infections based on acoustic, motor coordination, and phonemic patterns
Source: Sci Rep. 2023 Jan 28;13:1567. doi: 10.1038/s41598-023-27934-4 (PMC9884222; doi:10.1038/s41598-023-27934-4)
Supplement: Supplementary file 3 — Supplementary Information 3. [file 41598_2023_27934_MOESM3_ESM.pdf]

Title: Dissociating COVID-19 from other respiratory infections based on acoustic, motor coordination, and phonemic patterns

Authors:

Tanya Talkar[1,2*], Daniel M. Low[2,3], Andrew Simpkin[4,5], Satrajit Ghosh[2,3], Derek T. O'Keeffe[4], Thomas F. Quatieri[1,2]

Affiliations:

[1]MIT Lincoln Laboratory, Lexington, MA

[2]Speech and Hearing Bioscience and Technology, Harvard Medical School, Boston, MA
[3]MIT McGovern Institute for Brain Research, Cambridge, MA

[4]HIVE Lab, Lambe Institute, School of Medicine, National University of Ireland, Galway, Ireland

[5]School of Mathematical and Statistical Sciences, National University of Ireland, Galway, Ireland

*Tanya.Talkar@ll.mit.edu

Supplementary Material

Patient demographics and self-report of symptoms

Table S1 shows the self-reported demographics and symptoms that were gathered from each subject. The COVID-19 positive subjects (denoted by a 1 in the CovidStatus column) reported a greater number of symptoms than the COVID-19 negative subjects did. Only two subjects reported a history of smoking (denoted by a 2 in the Smoking column), while all others had no history of smoking.

Table S1. Patient demographics. Information for each subject analyzed in this study is provided in the following table. Age and gender were provided by the subject and COVID status was verified by a PCR test. All other symptoms were also reported by the subject, and the total number of symptoms reported per patient is provided in Symptom Count.

subjID	CovidStatus	Gender	Age	Smoking	Fever	Tiredness	Headache	Sore Throat	Breathing Difficulty	Mucal Cough	Dry Cough	Loss of Taste or	Stuffy Nose	Diarrhea	Chest Pain	Muscle Ache	Nausea	Symptom Count
0001	1	F	32	0	0	1	1	0	0	0	0	0	1	0	0	0	0	3
0002	1	M	38	0	1	1	1	1	0	0	1	0	0	0	0	0	0	5
0052	1	F	25	0	0	1	0	0	0	0	0	1	1	0	0	0	0	3
0054	1	F	33	0	0	1	1	0	0	0	0	1	1	0	0	0	0	4
0055	1	M	33	0	1	1	1	0	0	0	1	1	1	0	0	0	0	6
0056	1	F	32	0	0	1	1	1	0	0	1	1	1	0	0	0	0	6
0058	1	M	45	0	1	0	0	0	0	1	1	0	0	0	0	0	0	3
0062	1	M	24	0	1	1	1	0	0	0	0	1	1	0	0	0	0	5
0063	1	F	26	0	1	1	1	0	0	1	0	0	1	0	1	0	0	6
0079	1	M	27	0	0	0	0	0	0	0	0	0	0	0	0	0	0	0
0081	1	F	23	0	1	1	1	1	0	1	0	0	0	0	1	1	0	7
0091	1	M	21	0	0	0	0	0	0	0	0	0	0	0	0	0	0	0
0051	0	F	31	0	0	0	0	0	0	0	0	0	0	0	0	1	0	1
0053	0	M	59	0	0	0	0	0	0	0	0	0	0	0	0	1	0	1
0059	0	F	37	0	0	0	0	0	0	0	0	0	0	0	0	1	0	1
0060	0	F	31	0	0	0	0	0	0	0	0	0	0	0	0	1	0	1
0061	0	F	26	0	0	0	1	0	0	0	0	0	0	0	0	1	0	2
0068	0	F	23	0	0	0	0	0	0	0	0	0	0	0	0	1	0	1
0069	0	M	25	0	0	0	0	0	0	0	0	0	0	0	0	1	0	1
0071	0	F	22	2	0	0	0	0	0	0	0	0	0	0	0	1	0	1
0072	0	F	24	0	0	0	1	0	0	0	0	0	0	0	0	0	0	1
0073	0	F	31	0	0	0	0	0	0	0	0	0	0	0	0	1	0	1
0074	0	F	28	0	0	0	0	0	0	0	0	0	0	0	0	1	0	1
0075	0	M	30	0	0	0	0	0	0	0	0	0	0	0	0	1	0	1
0078	0	F	20	0	0	1	0	0	0	0	0	0	1	0	0	0	0	2
0087	0	F	30	0	0	0	0	0	0	0	0	0	0	0	0	0	0	0
0090	0	F	19	0	0	0	0	0	0	0	0	0	0	0	0	0	0	0

Split of data for training and testing of models

Figure S1 provides a description of how the bootstrapping procedure was completed with train-test splits of subjects. Each bootstrap iteration selected three subjects in the COVID-19 positive group and three subjects in the COVID-19 negative group to hold out completely as test subjects. This was done in both the case where the models were trained and tested on just the first day of data collection as well as the case where models were trained on Day 1 data and tested on Day 2 or Day 3 data. This ensured that there was no overlap in subject data between the train and test data, and therefore, there was no risk of subject identification in the models.

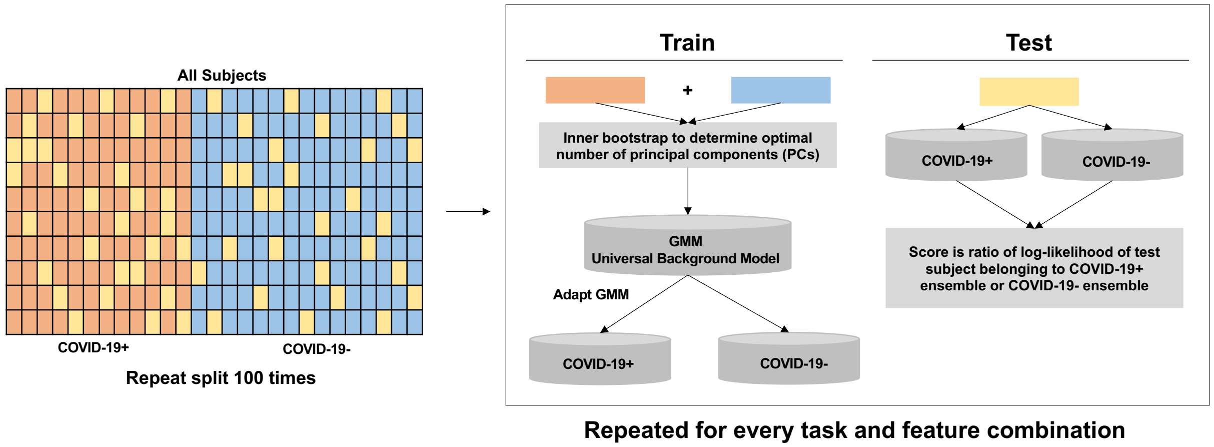

Figure S1. Depiction of model building. This describes how train/test splits were generated from the full dataset for each bootstrap iteration. No subject appeared in both the train and test splits, to avoid any speaker identification. The training set was used to determine an optimal number of principal components for dimensionality reduction and to generate an ensemble of GMMs as a universal background model, which were then adapted into COVID-19 positive and COVID-19 negative GMMs. These ensembles were used to determine the final likelihood of each held-out test subject belonging to each group, which was used to calculate the final AUC.

Computation of significance given multiple comparisons:

In this study, multiple models were compared across tasks, feature sets, and days of recording. To account for this, a harmonic mean p-value correction was utilized to determine which comparisons were significant. Wilcoxon rank-sum one-sided tests were computed across the bootstrap distributions of the AUCs all of the models generated to see whether one model's AUC was greater than another model's AUC. Using all of the p-values and comparisons across 606 models, the harmonic p-value was calculated. Any p-values that were less than the significant value of 0.05 and the harmonic p-value of 1.11×10^{-16} was considered significant, and therefore the AUCs of the two models were significantly different.

Table S2 shows the p-values of the models generated from acoustic features across all tasks by training and testing using the data from session one of recording. The cells in green are highlighted as those that are significant given the harmonic p-value. The p-values represent whether the model created from the task listed in the row is greater than that listed in the columns. For example, the model generated from univariate statistics of acoustic features derived from the Rainbow task had an AUC that was greater than that of the model generated from univariate statistics of acoustic features generated by Free Speech with a p-value of 3.60E-02.

Table S2. P-values for acoustic feature models for day one. The p-values for the one-sided Wilcoxon rank-sum tests for the models generated using univariate statistics of acoustic features, trained and tested on session one's data, are shown here. The ones that are significant given the calculated harmonic p-value are highlighted in green.

	**Free Speech**	**Count**	**Vowel**	**Rainbow**	**DDK**
**Free Speech**		0.941252	7.65E-05	0.964025	1.43E-07
**Count**	5.87E-02		8.22E-08	0.507369	1.80E-10
**Vowel**	1.00E+00	1		1	7.99E-02
**Rainbow**	3.60E-02	0.492631	1.82E-07		1.09E-10
**DDK**	1.00E+00	1	9.20E-01	1	

Table S3 shows an expanded version of Table S2, including the models trained on session one and tested on days 2 and 3 of collection. The cells in green are highlighted as those that are significant given the harmonic p-value. The p-values represent whether the model created from the task listed in the row is greater than that listed in the columns.

Table S3. P-values for all models based on acoustic feature time series. The p-values for the one-sided Wilcoxon rank-sum tests for all of the models generated using univariate statistics of acoustic feature time series are shown here. The ones that are significant given the calculated harmonic p-value are highlighted in green.

	Free Speech	Free Speech	Free Speech	Count	Count Day 2	Count Day 3	Vowel	Vowel Day 2	Vowel Day 3	Rainbow	Rainbow Day 2	Rainbow Day 3	DDK	DDK Day 2	DDK Day 3
**Free Speech**		9.12E-01	9.71E-01	0.941252	0.999932	0.999998	7.65E-05	8.77E-07	2.94E-13	0.964025	7.78E-01	1	1.43E-07	3.95E-01	7.76E-01
**Free Speech Day 2**	8.79E-02		9.75E-01	0.738209	0.999998	1	1.92E-07	5.93E-14	2.36E-17	0.908505	3.30E-01	1	1.02E-11	5.21E-02	4.27E-01
**Free Speech Day 3**	2.86E-02	2.50E-02		0.386346	0.980349	0.999967	3.61E-09	3.75E-16	8.66E-18	0.759115	2.53E-02	1	1.92E-13	1.05E-03	2.86E-02
**Count**	5.87E-02	2.62E-01	6.14E-01		0.951165	0.997401	8.22E-08	1.59E-09	2.75E-15	0.507369	1.15E-01	0.999991	1.80E-10	3.02E-02	2.48E-01
**Count Day 2**	6.81E-05	1.54E-06	1.97E-02	0.048835		0.998761	3.15E-12	3.34E-18	6.67E-18	0.077648	2.22E-07	0.999931	8.52E-15	1.32E-08	6.18E-04
**Count Day 3**	1.65E-06	8.94E-08	3.34E-05	0.002599	0.001239		3.92E-13	6.27E-18	2.38E-18	0.001002	1.53E-09	0.901392	6.13E-16	1.21E-10	9.74E-06
**Vowel**	1.00E+00	1.00E+00	1.00E+00	1	1	1		8.04E-01	7.46E-05	1	1.00E+00	1	7.99E-02	1.00E+00	1.00E+00
**Vowel Day 2**	1.00E+00	1.00E+00	1.00E+00	1	1	1	1.96E-01		1.28E-12	1	1.00E+00	1	9.78E-04	1.00E+00	1.00E+00
**Vowel Day 3**	1.00E+00	1.00E+00	1.00E+00	1	1	1	1.00E+00	1.00E+00		1	1.00E+00	1	9.90E-01	1.00E+00	1.00E+00
**Rainbow**	3.60E-02	9.15E-02	2.41E-01	0.492631	0.922352	0.998998	1.82E-07	1.96E-09	1.21E-14		3.06E-02	0.999943	1.09E-10	9.95E-03	1.11E-01
**Rainbow Day 2**	2.22E-01	6.70E-01	9.75E-01	0.884605	1	1	5.39E-08	2.13E-15	4.42E-17	0.969443		1	2.35E-12	4.93E-02	4.22E-01
**Rainbow Day 3**	3.68E-08	2.50E-11	2.09E-08	0.000009	0.000069	0.098608	6.60E-15	4.90E-18	2.11E-18	0.000057	1.32E-12		1.07E-16	2.20E-12	7.49E-08
**DDK**	1.00E+00	1.00E+00	1.00E+00	1	1	1	9.20E-01	9.99E-01	9.60E-03	1	1.00E+00	1		1.00E+00	1.00E+00
**DDK Day 2**	6.05E-01	9.48E-01	9.99E-01	0.969769	1	1	7.31E-06	2.40E-10	1.57E-15	0.990048	9.51E-01	1	1.28E-09		7.88E-01
**DDK Day 3**	2.24E-01	5.73E-01	9.71E-01	0.751959	0.999382	0.99999	9.08E-06	1.14E-08	5.29E-15	0.888659	5.78E-01	1	4.34E-09	2.12E-01	

Tables S4-S7 show the p-values for the phoneme-based models, utilizing various combinations of the phoneme-based features.

Table S4. P-values for phoneme models with all features. The p-values for the one-sided Wilcoxon rank-sum tests for all of the models generated using all of the phoneme-based features are shown here. The ones that are significant given the calculated harmonic p-value are highlighted in green.

	Free Speech	Free Speech Day 2	Free Speech Day 3	Count	Count Day 2	Count Day 3	Rainbow	Rainbow Day 2	Rainbow Day 3
**Free Speech**		5.10E-02	7.06E-12	3.60E-03	1.95E-02	1.41E-01	5.97E-02	1.09E-01	0.999991
**Free Speech Day 2**	0.948975		6.25E-16	1.01E-02	2.47E-01	8.21E-01	2.62E-01	6.92E-01	1
**Free Speech Day 3**	1	1.00E+00		1.00E+00	1.00E+00	1.00E+00	1.00E+00	1.00E+00	1
**Count**	0.996396	9.90E-01	6.70E-11		9.60E-01	9.87E-01	8.74E-01	9.91E-01	1
**Count Day 2**	0.980475	7.53E-01	6.56E-14	4.05E-02		9.30E-01	4.58E-01	8.25E-01	1
**Count Day 3**	0.859097	1.79E-01	4.84E-16	1.26E-02	7.01E-02		1.94E-01	3.08E-01	1
**Rainbow**	0.940297	7.38E-01	3.48E-09	1.26E-01	5.42E-01	8.06E-01		8.75E-01	1
**Rainbow Day 2**	0.891041	3.08E-01	1.69E-14	9.34E-03	1.75E-01	6.92E-01	1.25E-01		1
**Rainbow Day 3**	0.000009	1.33E-12	1.23E-17	3.80E-12	4.92E-11	2.26E-10	3.19E-10	6.08E-09	

Table S5. P-values for phoneme models using mean durations. The p-values for the one-sided Wilcoxon rank-sum tests for all of the models generated using the mean duration phoneme-based features are shown here. The ones that are significant given the calculated harmonic p-value are highlighted in green.

	Free Speech	Free Speech Day 2	Free Speech Day 3	Count	Count Day 2	Count Day 3	Rainbow	Rainbow Day 2	Rainbow Day 3
**Free Speech**		0.977863	3.63E-08	1.52E-03	1.58E-03	0.753642	1.56E-04	2.58E-07	0.353237
**Free Speech Day 2**	0.022137		8.05E-16	1.26E-08	1.55E-12	0.100708	9.56E-10	3.21E-14	0.000261
**Free Speech Day 3**	1	1		1.00E+00	1.00E+00	1	9.95E-01	6.47E-01	1
**Count**	0.998478	1	3.76E-04		5.50E-01	0.99976	1.40E-01	1.42E-03	0.998433
**Count Day 2**	0.998417	1	7.05E-05	4.50E-01		0.999924	1.22E-01	3.51E-05	0.999991
**Count Day 3**	0.246358	0.899292	1.17E-08	2.40E-04	7.55E-05		1.76E-04	3.49E-08	0.161595
**Rainbow**	0.999844	1	5.34E-03	8.60E-01	8.78E-01	0.999824		2.29E-02	0.999996
**Rainbow Day 2**	1	1	3.53E-01	9.99E-01	1.00E+00	1	9.77E-01		1
**Rainbow Day 3**	0.646763	0.999739	8.74E-12	1.57E-03	8.68E-06	0.838405	4.03E-06	2.51E-11	

Table S6. P-values for phoneme-based models using phoneme rate. The p-values for the one-sided Wilcoxon rank-sum tests for all of the models generated using the phoneme rate features are shown here. The ones that are significant given the calculated harmonic p-value are highlighted in green.

	Free Speech	Free Speech Day 2	Free Speech Day 3	Count	Count Day 2	Count Day 3	Rainbow	Rainbow Day 2	Rainbow Day 3
**Free Speech**		1.00E+00	9.83E-01	9.29E-01	1.00E+00	1.00E+00	0.999923	1	1
**Free Speech Day 2**	1.61E-09		1.62E-04	4.32E-07	1.42E-04	1.20E-01	0.054951	1	0.637157
**Free Speech Day 3**	1.74E-02	1.00E+00		1.47E-01	7.55E-01	9.86E-01	0.95921	1	0.999829
**Count**	7.11E-02	1.00E+00	8.53E-01		9.99E-01	1.00E+00	0.993917	1	0.999999
**Count Day 2**	2.82E-04	1.00E+00	2.45E-01	1.18E-03		9.70E-01	0.905397	1	0.999771
**Count Day 3**	9.39E-07	8.80E-01	1.39E-02	2.85E-04	3.04E-02		0.292869	1	0.883782
**Rainbow**	7.67E-05	9.45E-01	4.08E-02	6.08E-03	9.46E-02	7.07E-01		0.999999	0.965154
**Rainbow Day 2**	9.84E-15	1.42E-07	1.36E-11	3.98E-13	2.15E-12	4.72E-07	0.000001		0.000005
**Rainbow Day 3**	4.61E-09	3.63E-01	1.71E-04	1.22E-06	2.29E-04	1.16E-01	0.034846	0.999995	

Table S7. P-values for phoneme models using summary statistics. The p-values for the one-sided Wilcoxon rank-sum tests for all of the models generated using six summary statistic features are shown here. The ones that are significant given the calculated harmonic p-value are highlighted in green.

	Free Speech	Free Speech Day 2	Free Speech Day 3	Count	Count Day 2	Count Day 3	Rainbow	Rainbow Day 2	Rainbow Day 3
**Free Speech**		8.99E-01	0.999932	4.77E-01	1	8.48E-01	8.47E-03	1.70E-02	8.27E-01
**Free Speech Day 2**	1.01E-01		0.999953	1.27E-01	1	1.93E-01	2.81E-05	3.07E-05	8.16E-02
**Free Speech Day 3**	6.83E-05	4.73E-05		7.13E-05	0.871701	1.14E-05	2.78E-08	1.41E-08	7.85E-06
**Count**	5.23E-01	8.73E-01	0.999929		1	7.24E-01	2.35E-03	7.34E-03	8.27E-01
**Count Day 2**	3.27E-09	9.28E-13	0.128299	7.48E-12		1.08E-10	1.28E-13	2.60E-13	5.64E-12
**Count Day 3**	1.52E-01	8.07E-01	0.999989	2.76E-01	1		8.88E-05	4.16E-04	2.94E-01
**Rainbow**	9.92E-01	1.00E+00	1	9.98E-01	1	1.00E+00		1.89E-01	1.00E+00
**Rainbow Day 2**	9.83E-01	1.00E+00	1	9.93E-01	1	1.00E+00	8.11E-01		1.00E+00
**Rainbow Day 3**	1.73E-01	9.18E-01	0.999992	1.73E-01	1	7.06E-01	6.78E-06	6.31E-09	

Model performance represented in accuracy and F1 scores:

In addition to the area under the receiver operating characteristic (ROC) curve (AUC), the average accuracy and F1 scores of the models were calculated. These scores were determined by taking the optimal threshold on the ROC curve generated by the training data, and evaluating the confusion matrix at that optimal threshold using the test data. The following figures depict the accuracy and F1 scores for the categories of models generated. The trends in the accuracy and F1 scores generally mirrored the trends seen in the AUCs across different groups of models.

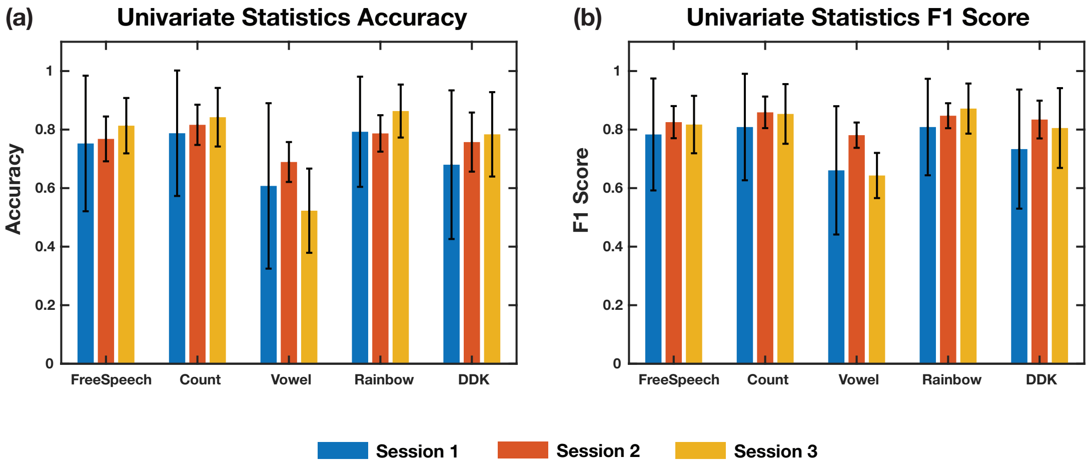

Figure S2. Model performance as accuracy and F1 score using low-level acoustic features. (a) Accuracy from models created using univariate statistics of acoustic and articulatory features. (b) F1 score from models created using univariate statistics of acoustic and articulatory features. The different bars in each task represent which of the recording days was used in the test data set. Error bars are the standard deviation of the statistic across all bootstrap iterations.

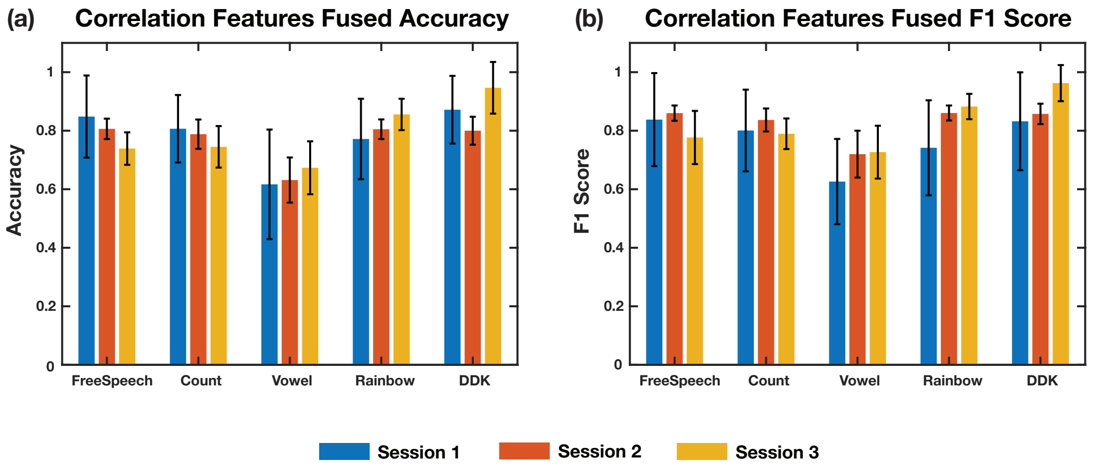

Figure S3. Fused model performance as accuracy and F1 score using correlation-based features. (a) Accuracy from a fused model utilizing all correlation-based features. (b) F1 score from a fused model utilizing all correlation-based features. The different bars in each task represent which of the recording days was used in the test data set. Error bars are the standard deviation of the statistic across all bootstrap iterations.

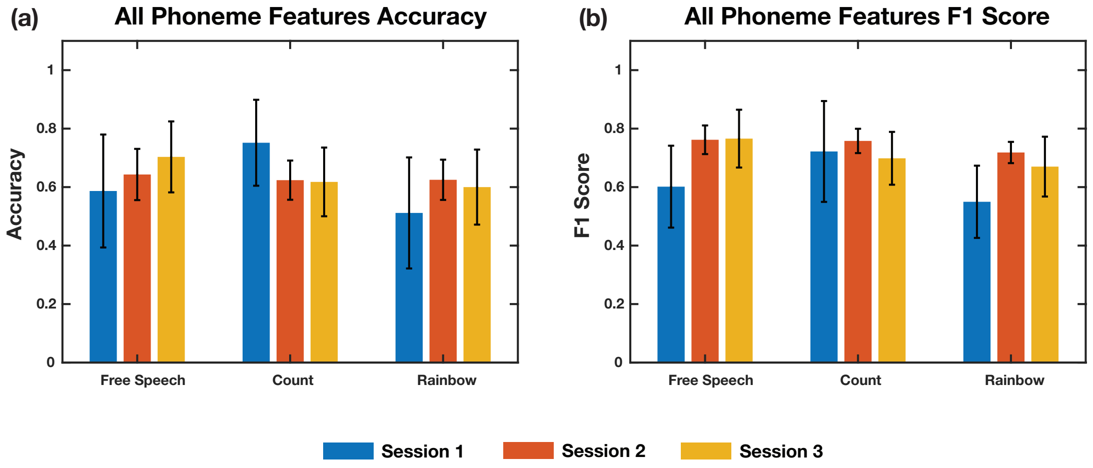

Figure S4. Model performance as accuracy and F1 score utilizing all phoneme-based features. (a) Accuracy from a fused model utilizing all phoneme-based features. (b) F1 score from a fused model utilizing all phoneme-based features. The different bars in each task represent which of the recording days was used in the test data set. Error bars are the standard deviation of the statistic across all bootstrap iterations.

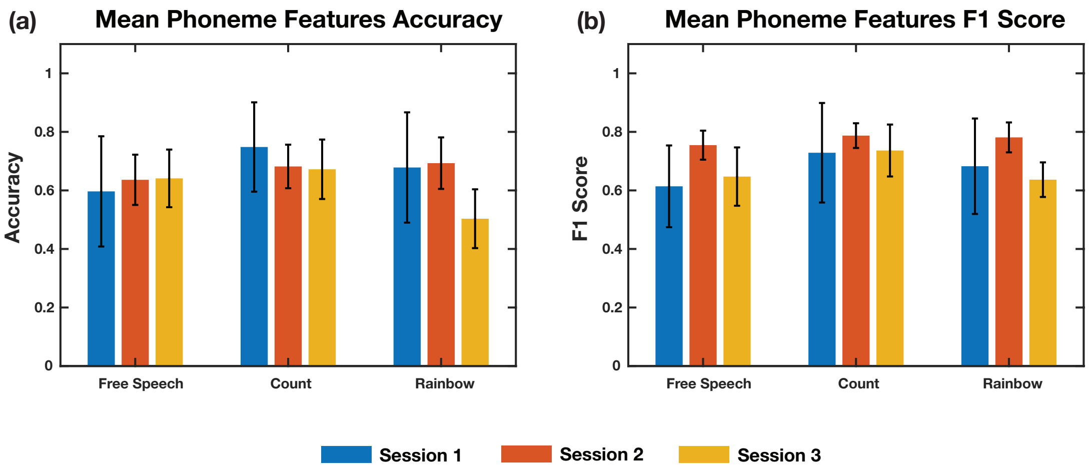

Figure S5. Model performance as accuracy and F1 score utilizing mean durations of phonemes. (a) Accuracy from a fused model utilizing the mean durations of all phonemes. (b) F1 score from a fused model utilizing the mean durations of all phonemes. The different bars in each task represent which of the recording days was used in the test data set. Error bars are the standard deviation of the statistic across all bootstrap iterations.

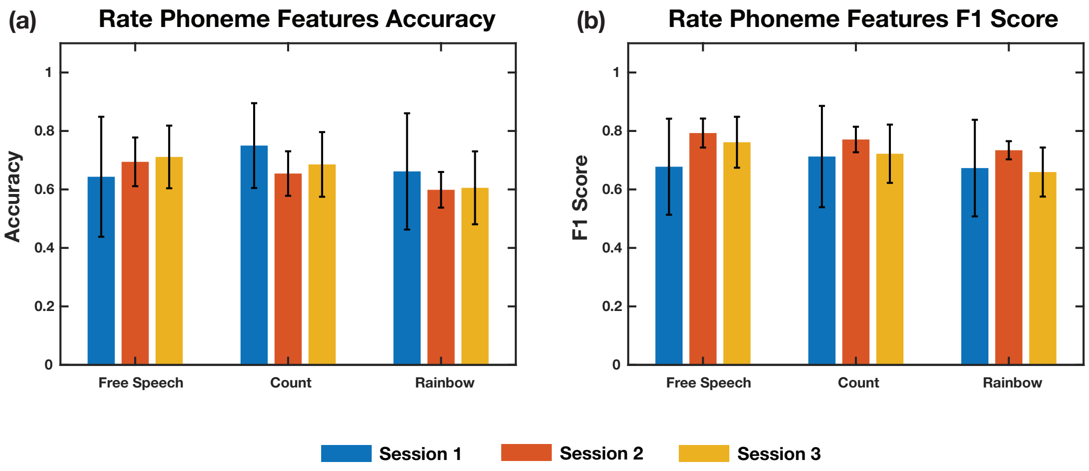

Figure S6. Model performance as accuracy and F1 score utilizing rates of phonemes. (a) Accuracy from a fused model utilizing the rates of all phonemes. (b) F1 score from a fused model utilizing the rates of all phonemes. The different bars in each task represent which of the recording days was used in the test data set. Error bars are the standard deviation of the statistic across all bootstrap iterations.

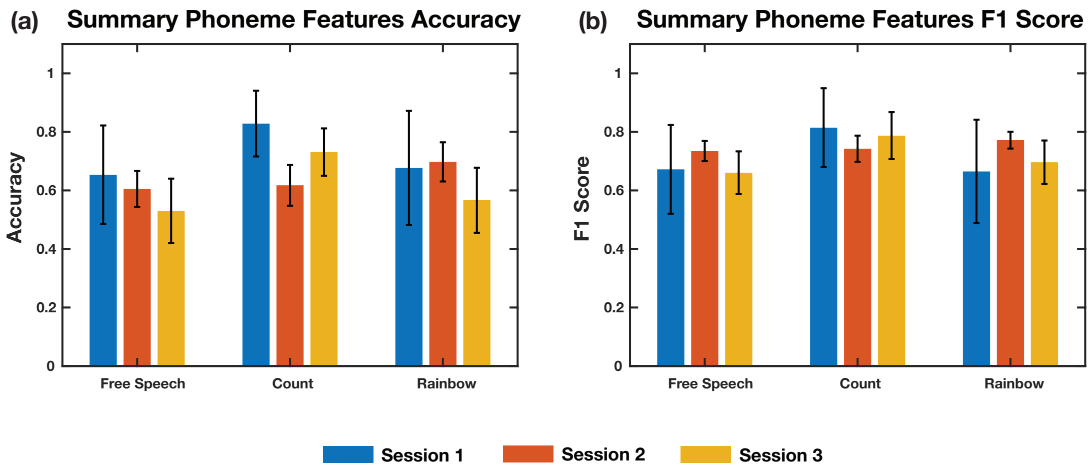

Figure S7. Model performance as accuracy and F1 score utilizing summary statistics of phonemes. (a) Accuracy from a fused model utilizing a set of six summary statistics of phoneme-based features. (b) F1 score from a fused model utilizing a set of six summary statistics of phoneme-based features. The different bars in each task represent which of the recording days was used in the test data set. Error bars are the standard deviation of the statistic across all bootstrap iterations.
